# Supplementary material for: KIF18A's neck linker permits navigation of microtubule-bound obstacles within the mitotic spindle
Source: Life Sci Alliance. 2019 Jan 17;2(1):e201800169. doi: 10.26508/lsa.201800169 (PMC6337737; doi:10.26508/lsa.201800169)
Supplement: Supplementary file 4 [file LSA-2018-00169_TableS1.doc]

Table S1. Summary of GFP-KIF18A^1-480^ WT (WT) and GFP-KIF18A^1-480^ sNL1 (sNL1) processive events. RK, rigor kinesin.

|  | **Uncorrected Run Length (µm)** | **Characteristic track length (µm)** | **Characteristic run length (µm)** | **Corrected Run Length (µm)** | **Velocity (µm/s)** | **N** |
| --- | --- | --- | --- | --- | --- | --- |
| **WT**  **No Tau** | 2.38 ± 2.01 | 4.74 ± 1.75 | 1.82 ± 0.37 | 2.35 ± 0.85 | 0.31 ± 0.14 | 190 |
| **WT + Tau** | 1.92 ± 1.38 | 4.51 ± 1.44 | 1.66 ± 0.32 | 2.08 ± 0.71 | 0.34 ± 0.15 | 194 |
| **SNL1 No Tau** | 0.96 ± 0.66 | 4.44 ± 0.78 | 0.70 ± 0.12 | 0.78 ± 0.21 | 0.50 ± 0.21 | 214 |
| **SNL1 + Tau** | 0.62 ± 0.31 | 4.61 ± 1.69 | 0.34 ± 0.07 | 0.36 ± 0.11 | 0.51 ± 0.19 | 207 |
| **WT**  **No RK** | 2.44 ± 2.19 | 4.81 ± 1.21 | 1.93 ± 0.44 | 2.50 ± 0.86 | 0.35 ± 0.18 | 55 |
| **WT +**  **RK** | 2.28 ± 1.91 | 4.67 ± 1.57 | 1.72 ± 0.39 | 2.19 ±0.72 | 0.37 ± 0.20 | 52 |
| **SNL1**  **No RK** | 0.89 ± 0.56 | 4.53 ± 1.15 | 0.75 ± 0.14 | 0.82 ±0.24 | 0.53 ± 0.24 | 54 |
| **SNL1 +**  **RK** | 0.73 ± 0.63 | 4.63 ± 0.76 | 0.58 ± 0.11 | 0.63 ±0.17 | 0.54 ± 0.21 | 51 |
